# Intestinal Permeability Associated with the Loss of Skeletal Muscle Strength in Middle-Aged and Older Adults in Rural Area of Beijing, China

**DOI:** 10.3390/healthcare10061100

**Published:** 2022-06-13

**Authors:** Cheng Li, Yaru Li, Nan Wang, Zhiwen Ge, Zhengli Shi, Jia Wang, Bingjie Ding, Yanxia Bi, Yuxia Wang, Zhongxin Hong

**Affiliations:** Department of Clinical Nutrition, Beijing Friendship Hospital, Capital Medical University, 95 Yongan Road, Xicheng District, Beijing 100050, China; lichengyys@126.com (C.L.); liyaru0225@126.com (Y.L.); wangnan0818@sina.com (N.W.); loggve@163.com (Z.G.); shiyaxi0531@sina.com (Z.S.); wangjia830106@163.com (J.W.); 13401174557@126.com (B.D.); biyanxia0407@163.com (Y.B.); mycat123@163.com (Y.W.)

**Keywords:** intestinal permeability, gut microbiota, handgrip strength, diamine oxidase, middle-aged and older adults

## Abstract

The association between intestinal permeability and sarcopenia remains unclear, and few studies have mentioned the relationship between intestinal permeability and skeletal muscle strength. The present cross-sectional community study was conducted in a rural area of Beijing to explore the association between intestinal permeability and handgrip strength (HGS) in middle-aged and older adults. Serum lipopolysaccharide (LPS), diamine oxidase (DAO) and *D*-lactate were detected to evaluate intestinal permeability. Gut microbiota (GM) and its potential interaction were also analyzed in the decision tree model. HGS was negatively correlated with DAO (*r* = −0.396, *p* < 0.01) in males. The negative association between HGS and DAO remained significant with the adjustment of covariates (*β* = −1.401, *p* < 0.05). Serum DAO and LPS were both negatively associated with HGS in middle-aged and older males, with the significant interactions of GM in the decision tree model, and *D*-lactate showed a negative association with HGS in females. Therefore, intestinal permeability was associated with the loss of skeletal muscle strength in middle-aged and older adults, and serum DAO may be a novel predictor for the loss of skeletal muscle strength in middle-aged and older males.

## 1. Introduction

Sarcopenia, closely associated with frailty and disability, has been widely recognized as a progressive and generalized skeletal muscle disease involving skeletal muscle recession and dysfunction in recent years [1,2]. Diagnostic criteria have been recommended and updated by multiple expert panels in the last decade, such as the European Working Group on Sarcopenia in Older People (EWGSOP) [3] and the Asian Working Group for Sarcopenia (AWGS) [4]. Although there are many different versions of diagnostic criteria, handgrip strength (HGS), a classical surrogate indicator of skeletal muscle strength, has been widely recognized as an indispensable indicator in the diagnosis of sarcopenia [5,6].

Compared with skeletal muscle mass, the measurement of HGS is convenient and inexpensive, and it has been advised for routine measurement for hospital practice and community healthcare [5,7]. Previous studies indicated that HGS may be associated with multiple health outcomes in multiple populations, such as hospital stays in rehabilitation inpatients aged 70 years and older [8], quality of daily life in adults aged 65 years and older [9] and all-cause mortality of people from diverse economic and sociocultural backgrounds with a median age of 50 years [10]. A significantly positive association between lower HGS and all-cause mortality was reported in a perspective cohort study with half of a million UK Biobank participants aged 40–69 years [11]. A systematic review and meta-analysis involving more than 100,000 older adults aged 65 years and older found that low HGS was associated with worsening activities of daily living, indicating that HGS may be a meaningful predicator for activities of daily living [12]. Furthermore, a recent umbrella review of systematic reviews with meta-analyses of observational studies including multiple populations found that HGS was a useful indicator for general health status and disability, specifically for early all-cause and cardiovascular mortality [13]. The above studies showed intensive associations between HGS and general health outcomes, and they indicated the significantly predicating value of HGS in multiple health outcomes in middle-aged and older adults.

Aging, systematic inflammation, insulin resistance, and inadequate nutrition were considered as the dominant inducements of skeletal muscle recession and sarcopenia [1,14]. Recently, with the rising hypothesis of gut microbiota (GM)–muscle axis, GM has been considered as a novel modulator of skeletal muscle health [15]. GM dysbiosis may attenuate intestinal health and then trigger systemic inflammation [16] and insulin resistance [17], increasing the risk of muscle recession [18]. Due to the change of intestinal permeability, the micronutrients and metabolites derived by GM, such as lipopolysaccharide (LPS) [17] and short-chain fatty acids [19], could reach skeletal muscle and other tissues; then, they regulate skeletal muscle health via multiple pathways [15]. An animal study showed that germ-free mice were fatigable and with attenuated muscle strength, and microbiota transplantation from the pathogen-free mice could increase muscle mass and reduce muscle atrophy in the germ-free mice [20]. *Lactobacillus* and *Bifidobacterium* supplements notably enhanced both muscle strength and endurance capacity in aged mice [21]. The supplement of kefir reduced the *Firmicutes*/*Bacteroidetes* ratio and significantly increased the grip strength of mice [22]. A comparison study between sedentary older adults (70–85 years) with different levels of physical function revealed that higher levels of *Prevotellaceae*, *Prevotella*, *Barnesiella*, and *Barnesiella intestinihominis* were found in the older adults with higher levels of physical function and leg muscle strength [23]. However, no significant alimentation of skeletal muscle strength was observed in the older adults between 60 and 75 years with a 3-month supplement of symbiotic in the previous pilot study [24]. Although the GM–muscle axis has attracted widespread attention, the research evidence involving GM and HGS is still limited. Furthermore, most of the previous studies only focused on the association between GM and skeletal muscle strength; the potential relationship between intestinal permeability and HGS was rarely mentioned. Compared with GM, intestinal permeability may have a more direct effect on skeletal muscle strength. Therefore, in addition to considering the effect of GM, it may be more practical to explore the relationship between intestinal permeability and skeletal muscle strength.

Given the hypothesis of the GM–muscle axis and the potential association between GM and sarcopenia, we explored the associations among intestinal permeability, GM, and skeletal muscle strength. In the present study, HGS and typical biomarkers of intestinal permeability, including LPS, diamine oxidase (DAO), and *D*-lactate were, respectively, assessed in the middle-aged and older rural residents of Zhangfang village, Fangshan district, Beijing. The association between intestinal permeability and HGS was analyzed. Furthermore, with the potential interaction of GM, the association among intestinal permeability, GM and HGS was also assessed in the decision tree model in the present study.

## 2. Participants, Materials and Methods

### 2.1. Participants and Study Design

The present study was conducted in Zhangfang village, Fangshan district, Beijing in 2019. Pre-investigation and participants recruitment were conducted from May to June. On-field investigations, including a face-to-face questionnaire survey, anthropometric measurements, and sample collections, were conducted within a 2-day period in late June. Laboratory testing was conducted from July to September. Rural residents in Zhangfang village were recruited in the present study. Residents aged 45–64 years were recruited in this study as middle-aged adults, while residents aged 65 years and older were recruited as older adults. Individuals with the following conditions were excluded: self-reported gastrointestinal disease; self-reported respiratory infection; self-reported stroke, coronary artery disease or other several cardiovascular diseases; and self-reported intake history of antibiotics or probiotics in the past month. This study was conducted in accordance with the Declaration of Helsinki, and it was approved by the Ethics Committee of Beijing Friendship Hospital, Capital Medical University (Ethics number: 2019-P2-097-01).

### 2.2. Anthropometric Measurements

All the anthropometric measurements were conducted by trained doctors and clinical dietitians on the same day. Body mass and height were measured at once, respectively. Body mass index (BMI) was calculated as body mass (kg)/height^2^ (m^2^). Percent of body fat (PBF) and appendicular skeletal muscle mass were detected by a bioimpedance device (Inbody 770, Biospace, Seoul, Korea). Appendicular skeletal muscle mass index (ASMI) was calculated as appendicular skeletal muscle mass (kg)/height (m^2^) [6]. HGS (kg) was assessed in a standing position using an electronic dynamometer (CAMRY EH101, Xiangshan, Zhongshan, Guangdong, China). Both hands were assessed, and the maximum strength was recorded as HGS of the participant. Relative HGS was adjusted by BMI (HGSB) or body mass (HGSW), respectively.

### 2.3. Intestinal Permeability

Blood samples were collected by registered nurses in the morning before anthropometric measurements. All the blood samples were obtained in the morning after a 12 h fast; then, they were centrifuged within 2 h after blood collection and stored at −80 °C. LPS, DAO and *D*-lactate were measured as the indicators of intestinal permeability. Serum LPS was measured by ELISA using a microplate photometer (Multiskan EX, Thermo Fisher Scientific, Inc. Waltham, MA, USA); serum DAO and *D*-lactate were measured by the colorimetric method using a visible light spectrophotometer (721G, Yili Analytical Instrument, Shanghai, China).

### 2.4. Gut Microbiota Analysis

The collection of fecal samples was conducted by participants under the guidance of trained doctors within a 2-day period. On the first day, the doctor explained how to collect fecal samples for all the participants and distributed sampling tubes to the participants. On the second day, the fecal samples were examined and collected by the trained doctors before blood collection and anthropometric measurements. A commercial analysis kit (OMEGA Mag-bind universal pathogen 96 kit) was used to extract DNA following the manufacturer’s instructions. V3–V4 hypervariable regions of the bacteria 16s rDNA were then amplified. The purified amplicons were fluorescently quantified and further sequenced on the Illumina Miseq high-throughput sequencing platform with Miseq reagent kit V3-V4 and PhiX control V3–V4.

### 2.5. Potential Covariates

Potential covariates such as gender, age, daily exercise level and disease information were obtained by trained doctors and clinical dietitians through a face-to-face questionnaire survey after blood extraction and anthropometric measurements. Gender and age information were extracted from the identification card of the participants. Daily exercise level was determined by the type of exercise that the respondents did most in their daily life. The diagnosis information of hypertension and type 2 diabetes (T2Ds) was offered and self-reported by participants.

### 2.6. Statistical Analysis

Data of different items of the present study are collected and sorted, respectively, by different trained researchers and then checked and analyzed by two statistical experts. Quantitative data were expressed as mean ± standard deviation; comparisons between two groups were performed by *t*-test or Wilcoxon test, according to the data distribution. The Shapiro–Wilk test and Q-Q plot were used for the normality test of quantitative data. Categorical data were expressed as *n* (%) and were compared by chi-squared tests or Fisher’s exact test. Pearson correlation or Spearman rank correlation was performed according to the distribution of data. Association between intestinal permeability and HGS was analyzed by multiple linear regression analysis with and without the adjustment of relevant covariates. Model 1 was a crude model; model 2 was adjusted by age, BMI, ASMI, and PBF; model 3 was adjusted by age, BMI, ASMI, PBF, daily exercise level, hypertension, and T2Ds. The association between intestinal permeability, gut microbiota and HGS were analyzed by the decision tree model. The classification and regression tree (CRT) method was used in the model. The maximum tree depth in the model was set as 3, the minimum cases in the father node was set as 4, and the minimum cases in the child node was set as 2. All the models were performed with cross-validation. All the statistical analyses were performed using SAS 9.4 (SAS Institute Inc., Cary, NC, USA) and SPSS 22 (IBM SPSS, Inc., Chicago, IL, USA). *p* < 0.05 was considered as statistically significant.

## 3. Results

### 3.1. Participants Characteristics

A total number of 92 middle-aged and older adults (47 males and 45 females) were finally analyzed in the present study. General characteristics of the participants are presented in Table 1. There were no significant differences between males and females regarding BMI, the prevalence of hypertension and T2Ds. Meanwhile, the PBF values of males were significantly lower than those of females (*p* < 0.01), while the ASMI values of males were significantly higher than those of females. The skeletal muscle strength of males was significantly higher than that of females with and without adjustment of body mass and BMI (*p* < 0.01). Subgroup analyses by age are presented in Tables S1 and S2.

### 3.2. Correlation between Intestinal Permeability and Skeletal Muscle Strength

Different correlations were observed among the three indicators of intestinal permeability (DAO, LPS, *D*-lactate) and skeletal muscle strength (Table 2). Significantly negative correlation was observed between serum DAO and HGS (*r* = −0.396, *p* < 0.01). Similar correlation was observed between DAO and HGS adjusted by BMI (HGSB, *r* = −0.307, *p* < 0.05), while the significantly negative correlation between DAO and HGS was attenuated and disappeared with the adjustment of body mass (HGSW, *r* = −0.263, *p* > 0.05). No significant correlation was observed between DAO and HGS in females, with or without adjustment. In addition, no significant correlation was observed among the other indicators of intestinal permeability (LPS and *D*-lactate) and HGS, with or without adjustment. Subgroup analyses by age are presented in Table S3.

### 3.3. Association between Serum DAO and the Loss of Skeletal Muscle Strength in Middle-Aged and Older Adults

Association between serum DAO and the loss of skeletal muscle strength in middle-aged and older males was assessed in multiple linear regression model and is presented in Table 3. A significantly negative association between serum DAO and HGS was observed in the crude model (*β* = −1.862, 95% *CI* = −3.158~−0.566, *p* < 0.01). Although the negative association was attenuated with multiple adjustments, serum DAO still showed a significantly negative association with HGS (*β* = −1.401, 95% *CI* = −2.566~−0.235, *p* < 0.05) with the adjustments of potential covariates, including age, BMI, ASMI, PBF, daily exercise level, hypertension and T2Ds. In addition, significant associations among DAO and HGSW (*β* = −0.017, 95% *CI* = −0.032~−0.001, *p* < 0.05), HGSB (*β* = −0.058, 95% *CI* = −0.105~−0.012, *p* < 0.05) were also observed in the adjusted multiple linear regression models. Meanwhile, no significant association was observed between skeletal muscle strength and serum DAO in middle-aged and older females (Table 4). Subgroup analyses of the association between DAO and HGS by age are presented in Tables S4 and S5. No significant association was observed among LPS, *D*-lactate and HGS (Tables S6–S9).

### 3.4. Association and Interaction among Indicators of Intestinal Permeability, GM and Skeletal Muscle Strength in Males and Females

**Figure 1:**
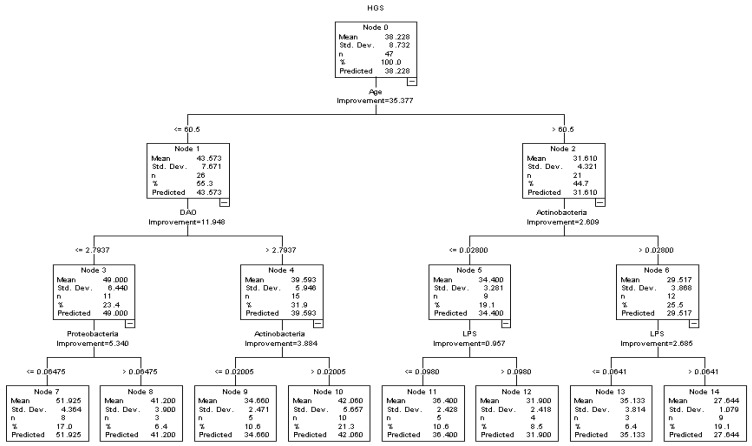
Associations among intestinal permeability, GM and HGS in middle-aged and older males in the decision tree model.

**Figure 2:**
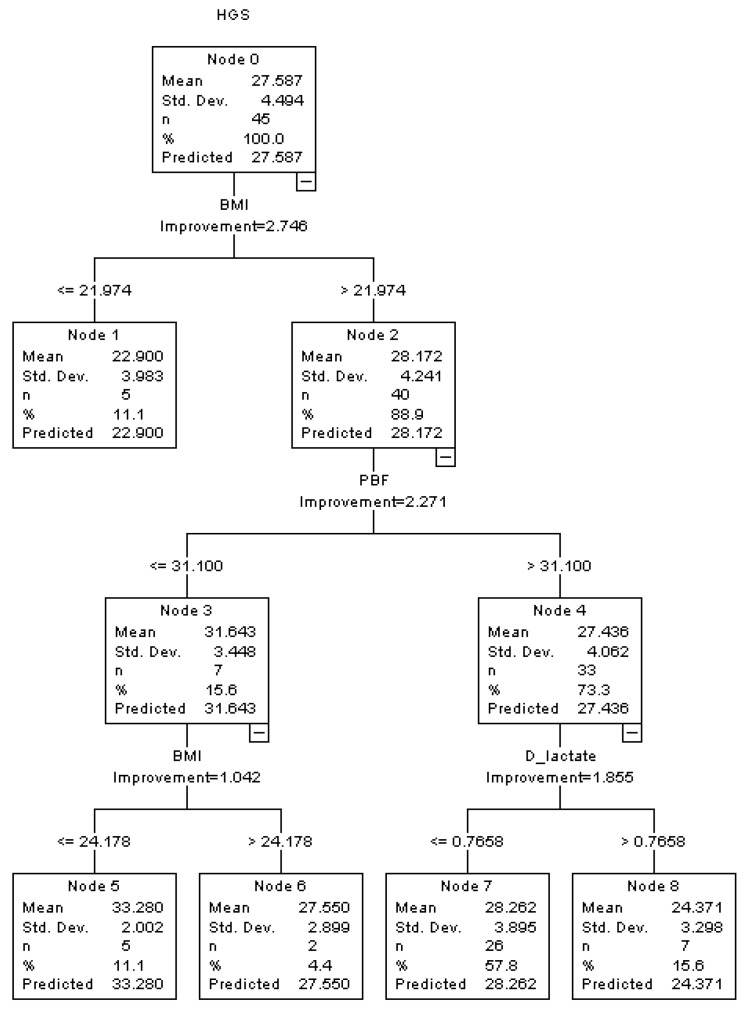
Associations among intestinal permeability, GM and HGS in middle-aged and older females in the decision tree model.

A decision tree model was used to explore the association and interaction among the typical indicators of intestinal permeability (DAO, LPS, *D*-lactate), dominant GM (*Actinobacteria*, *Bacteroidetes*, *Firmicutes*, *Proteobacteria*) and skeletal muscle strength in middle-aged and older males (Figure 1) and females (Figure 2). Potential covariates, including age, BMI, PBF, ASMI, daily exercise level, hypertension and T2Ds were all considered and analyzed in the decision tree model. In the three-depth tree, a total number of 15 nodes and 8 terminal nodes were observed, which was grown by the CRT method in Figure 1. Age, DAO and *Actinobacteria* ranked in the first two depths. In line with the previous result, a significantly negative association between DAO and HGS was observed in the decision tree model. In the younger participants (age ≤ 60.5 years), compared with the males with lower levels of DAO (≤ 2.794 U/L) and higher HGS (49.000 ± 6.440 kg), males with higher levels of DAO (>2.794 U/L) were accompanied with lower levels of HGS (39.593 ± 5.946 kg). In addition, a significantly negative association was observed between LPS and HGS in the older participants (age > 60.5 years); with the interaction between LPS and *Actinobacteria*, participants with higher levels of LPS showed lower HGS than the participants with lower levels of LPS. With the interactions of age and DAO, *Proteobacteria* showed a significantly negative association with HGS in the younger participants (age ≤ 60.5 years), while *Actinobacteria* showed a positive association with HGS (Figure 1). The associations among intestinal permeability, GM, and relative HGS (HGSW, HGSB) in males were also explored in the decision tree models and are, respectively, presented in the Supplemental Figures (Figures S1 and S2).

A decision tree model was also used to explore the association and interaction among the typical indicators of intestinal permeability (DAO, LPS, *D*-lactate), dominant GM (*Actinobacteria, Bacteroidetes, Firmicutes, Proteobacteria*) and HGS in middle-aged and older females (Figure 2). In females, BMI and PFB rank in the first two depths in the decision tree model. In the subgroup of participants with higher BMI and PBF, *D*-lactate showed a negative association with HGS. In females, higher levels of *D*-lactate (>0.766 mM) were accompanied by lower levels of HGS (24.371 ± 3.298 kg). However, no significant association was observed among DAO, LPS, dominant GM and HGS. The associations among intestinal permeability, GM and relative HGS (HGSW, HGSB) in females are presented in the Supplemental Figures (Figures S3 and S4).

## 4. Discussion

In the present study, we assessed intestinal permeability and HGS in the middle-aged and older adults in a rural area of Beijing, China. A significantly negative association was observed between serum DAO, a typical indicator of intestinal permeability, and HGS in middle-aged and older males. Meanwhile, no significant association was found between serum DAO and HGS in middle-aged and older females. To the best of our knowledge, the association between DAO and HGS in middle-aged and older adults was discussed for the first time in the present study. Relevant results indicated the importance of intestinal integrity in maintaining skeletal muscle strength in middle-aged and older adults. Furthermore, interactions among intestinal permeability, GM and HGS were also explored in the present study. DAO, *Proteobacteria* and LPS were negatively associated with HGS in middle-aged and older males. Meanwhile, *D*-lactate showed a potential negative association with HGS in the middle-aged and older females, and the association was affected by BMI and PBF. Compared with LPS and *D*-lactate, DAO may be the most valuable intestinal predictor of the loss of skeletal muscle strength in middle-aged and older males. Relevant results provided scientific evidence for the management of intestinal health and the prevention of skeletal muscle recession.

### 4.1. Intestinal Permeability and Skeletal Muscle Strength: The Potential Predicative Value of DAO for Sarcopenia?

In recent years, with the rising attention of the GM–muscle axis in muscle recession, several studies have been conducted to explore the association between GM and sarcopenia, but few of them discussed the associations and interactions among intestinal permeability, GM, and muscle health. Previous studies found that aging-induced impairment of the intestinal barrier may be a trigger for microbial translocation and low-grade chronic inflammation [25,26], which may increase the risk of skeletal muscle recession and sarcopenia [27]. A recent study found that butyrate could exert protect effects on skeletal muscle atrophy by enhancing the intestinal barrier function in mice [28]. Those studies indicated the importance of the intestinal barrier for sarcopenia. However, the association between intestinal barrier and sarcopenia in the community-dwelling population was still unclear. Serum levels of DAO, LPS and *D*-lactate, which were typical markers of intestinal barrier function, were selected in the present study to access the intestinal permeability of community-dwelling people. Partially in line with the previous studies, a significant association between the impairment of intestinal barrier and skeletal muscle recession was also found in the present study. In the present study, serum DAO was negatively correlated with HGS in males (Table 2). In addition, serum DAO showed a significantly negative association with the skeletal muscle strength of middle-aged and older males in the multiple linear regression models with and without adjustments (Table 3). DAO, an intracellular enzyme dominantly located in the mucosal villous epithelial cells, mainly catalyzed the oxidation of diamines [29,30]. Following the damage of intestinal mucosal cells, the translocation of intracellular DAO led to an increase in circulating DAO, reflecting the change of structural status and intestinal permeability in the small intestine [30]. At the same time, LPS and *D*-lactate could also penetrate the intestinal barrier and lead to the increase in circulating LPS and *D*-lactate [31,32]. The association between the impairment of intestinal barrier and sarcopenia indicated that several serum indicators of intestinal permeability may be the potential indicator of skeletal muscle recession. However, the association between DAO and skeletal muscle function was rarely mentioned. To the best of our knowledge, this was the first study to discuss the relationship between serum DAO and HGS in middle-aged and older adults.

In contrast to DAO, the potential relationship between LPS and skeletal muscle recession has been discussed extensively. A previous study found that circulating LPS could induce TNF-α and IL-6 and then lead to the muscle wasting in mice [33]. In addition, compared with younger individuals, older individuals showed higher levels of plasma LPS and the expression of TLR4 in skeletal muscle with the lower level of insulin sensitivity [34]. The adverse effects of LPS on skeletal muscle metabolism revealed that after entering the circulation system, LPS could induce muscle recession through multiple pathways [31]. Meanwhile, the association between circulating LPS and human skeletal muscle strength was rarely mentioned. In the present study, an interaction between GM and circulating LPS was found in males. In the older males, LPS showed a significantly negative association with HGS with the interaction of *Actinobacteria* (Figure 1). In females with higher BMI and PBF, *D*-lactate, another indicator of intestinal permeability, showed a significantly negative association with HGS in the decision tree model (Figure 2). Those results reflected the potential predictive value of intestinal permeability for muscle recession and sarcopenia, and they further indicated that DAO may be a more sensitive indicator of intestinal permeability and muscle recession in males.

### 4.2. Association and Interaction among GM, Intestinal Permeability, and Sarcopenia

Although the hypothesis of the GM-muscle axis has been raised in recently years, the casual relationship between GM and muscle recession is still unclear. Previous studies indicated that microbiota dysbiosis could increase intestinal permeability and then trigger systemic inflammation and insulin resistance, increasing the risk of muscle wasting and dysfunction [15,16]. The supplement of kefir reduced the *Firmicutes/Bacteroidetes* ratio and increased the grip strength of mice [22]. In an observation study including 23 of the oldest people, who ranged from 70 to 100 years, a significant reduction in *Bacteroides/Prevotella* was observed in the older adults with high frailty scores [35]. Another comparative trial showed that aerobic training could increase the intestinal abundance of intestinal *Bacteroides* and improved gait speed in older sedentary women [36]. However, the evidence about GM and skeletal muscle strength was still limited. A previous animal study found that a diet low in fermentable fiber was associated with larger numbers of *Firmicutes*, *Actinobacteria*, and smaller numbers of *Bacteroidetes* in mice. In addition, compared with the mice with a high fermentable fiber diet, the mice with a low fermentable fiber diet showed lower muscle mass and muscle endurance [37]. In the present study, the negative association was observed between *Actinobacteria* and HGS in older males aged over 60.5 years (Figure 1). However, the association between GM and muscle recession was still inconsistent in the present studies. Similar with the previous studies, the present study showed several significant associations among GM and HGS; besides, our study also found significant interactions among indicators of intestinal permeability and GM. The interaction among intestinal permeability and GM indicated the importance of intestinal health in the treatment and management of muscle recession in middle-aged and older adults, and it may partially explain the inconsistent associations among GM and sarcopenia in several previous studies.

There are several limitations in the present study. First, there are multiple detectable indicators of skeletal muscle strength and physical performance, including HGS, gait speed and quadriceps muscle strength. Only HGS was conducted in the present study. More indicators of skeletal muscle strength and physical function should be conducted and analyzed in the future study. Second, more longitudinal studies are required to validate the relationship between intestinal permeability and skeletal muscle strength as well as determine the predictive value of DAO for the muscle recession. Third, the population of the present study is rural community-dwelling residents, whose dietary patterns and daily physical activity differ significantly from urban residents and hospitalized patients. Meanwhile, those differences may have an impact on intestinal barrier function and skeletal muscle health. The association between DAO and skeletal muscle strength needs to be discussed in those specific populations in future studies.

## 5. Conclusions

In the present study, a significant association was observed between intestinal permeability and HGS the in middle-aged and older adults from the rural community of Beijing, China. Compared with LPS and *D*-lactate, DAO may be a novel predictor for the loss of HGS in middle-aged and older males. GM and intestinal permeability may have a significant interaction on skeletal muscle strength, and the association between DAO and HGS may be affected by GM composition. In future studies, it is necessary to further explore the potential mechanisms underlying the interaction between GM composition and intestinal permeability. Furthermore, the association between intestinal barrier function and skeletal muscle metabolism needs to be further explored in urban residents and hospitalized patients.

## Figures and Tables

**Table 1:** General characteristics.

Characteristic	Total	Male	Female	
	*n* = 92	*n* = 47	*n* = 45	*p* Value
Age (y)	58.3 ± 6.6	60.1 ± 7.4	56.4 ± 5.1	0.006
Body mass (kg)	68.3 ± 10.5	71.4 ± 10.9	65.0 ± 9.1	0.003
BMI (kg/m^2^)	25.4 ± 3.2	25.1 ± 3.1	25.7 ± 3.4	0.453
PBF (%)	31.0 ± 8.7	25.9 ± 5.7	36.3 ± 8.2	<0.001
ASMI (kg/m^2^)	7.1 ± 1.0	7.5 ± 1.2	6.7 ± 0.7	<0.001
HGS (kg)	33.0 ± 8.8	38.2 ± 8.7	27.6 ± 4.5	<0.001
HGSW	0.5 ± 0.1	0.5 ± 0.1	0.4 ± 0.1	<0.001
HGSB	1.3 ± 0.4	1.5 ± 0.3	1.1 ± 0.2	<0.001
Daily exercise level				
Light (*n*, %)	2 (2.2)	2 (4.3)	0 (0.0)	0.002
Moderate (*n*, %)	31 (33.7)	22 (46.8)	9 (20.0)	
Vigorous (*n*, %)	57 (61.9)	21 (44.6)	36 (80.0)	
No response (*n*, %)	2 (2.2)	2 (4.3)	0 (0.0)	
Chronic diseases				
Hypertension (*n*, %)	44 (47.8)	25 (53.2)	19 (42.2)	0.292
T2Ds (*n*, %)	17 (18.5)	9 (19.2)	8 (17.8)	0.866

Quantitative data are shown as mean ± SD, categorical data are shown as *n* (%). Abbreviations: BMI—Body mass index; PBF—Percent of body fat; ASMI—Appendicular skeletal muscle mass index; HGS—Handgrip strength; HGSW—Handgrip strength adjusted by body mass; HGSB—Handgrip strength adjusted by BMI; T2Ds—Type 2 diabetes.

**Table 2:** Correlations among indicators of intestinal permeability and skeletal muscle strength.

Indicator of	Gender	HGS		HGSW		HGSB	
Intestinal Permeability		*r*	*p*	*r*	*p*	*r*	*p*
DAO	Male	−0.396	0.006	−0.263	0.074	−0.307	0.036
	Female	0.057	0.710	−0.027	0.859	0.025	0.870
LPS	Male	0.041	0.783	0.084	0.573	0.038	0.799
	Female	0.199	0.189	0.115	0.452	0.102	0.504
*D*-lactate	Male	−0.089	0.551	−0.186	0.211	−0.210	0.157
	Female	−0.083	0.589	0.060	0.697	0.088	0.567

Abbreviations: DAO—Diamine oxidase; LPS—Lipopolysaccharide; HGS—Handgrip strength; HGSW—Handgrip strength adjusted by body mass; HGSB—Handgrip strength adjusted by BMI.

**Table 3:** Associations among serum DAO and skeletal muscle strength in middle-aged and older males.

Model	HGS				HGSW				HGSB			
	*β* Value	95% *CI*		*p* Value	*β* Value	95% *CI*		*p* Value	*β* Value	95% *CI*		*p* Value
Model 1	−1.862	−3.158	−0.566	0.006	−0.015	−0.032	0.002	0.074	−0.058	−0.111	−0.004	0.036
Model 2	−1.206	−2.226	−0.186	0.022	−0.015	−0.028	−0.001	0.037	−0.051	−0.092	−0.010	0.015
Model 3	−1.401	−2.566	−0.235	0.020	−0.017	−0.032	−0.001	0.034	−0.058	−0.105	−0.012	0.016

Model 1: Crude model. Model 2: Adjusted by age, BMI, ASMI, PBF. Model 3: Adjusted by age, BMI, ASMI, PBF, daily exercise level, hypertension and T2Ds. Abbreviations: HGS—Handgrip strength; HGSW—Handgrip strength adjusted by body mass; HGSB—Handgrip strength adjusted by BMI.

**Table 4:** Associations among serum DAO and skeletal muscle strength in middle-aged and older females.

Model	HGS				HGSW				HGSB			
	*β* Value	95% *CI*		*p* Value	*β* Value	95% *CI*		*p* Value	*β* Value	95% *CI*		*p* Value
Model 1	0.167	−0.730	1.064	0.710	−0.001	−0.018	0.015	0.859	0.004	−0.041	0.049	0.870
Model 2	−0.203	−1.219	0.814	0.689	0.000	−0.016	0.015	0.954	−0.005	−0.046	0.035	0.791
Model 3	−0.292	−1.343	0.759	0.577	−0.004	−0.020	0.013	0.646	−0.010	−0.052	0.033	0.650

Model 1: Crude model. Model 2: Adjusted by age, BMI, ASMI, PBF. Model 3: Adjusted by age, BMI, ASMI, PBF, daily exercise level, hypertension and T2Ds. Abbreviations: HGS—Handgrip strength; HGSW—Handgrip strength adjusted by body mass; HGSB—Handgrip strength adjusted by BMI.

## Data Availability

Data are available from the corresponding author upon reasonable request due to privacy restrictions.

## Supplementary Materials

The following supporting information can be downloaded at: https://www.mdpi.com/article/10.3390/healthcare10061100/s1, Table S1: Subgroup analysis of general characteristics of males by age; Table S2: Subgroup analysis of general characteristics of females by age; Table S3: Correlations among indicators of intestinal permeability and skeletal muscle strength in middle-aged and older adults; Table S4: Associations among serum DAO and skeletal muscle strength in middle-aged adults; Table S5: Associations among serum DAO and skeletal muscle strength in older adults; Table S6: Associations among serum LPS and skeletal muscle strength in middle-aged and older males; Table S7: Associations among serum LPS and skeletal muscle strength in middle-aged and older females; Table S8: Associations among serum *D*-lactate and skeletal muscle strength in middle-aged and older males; Table S9: Associations among serum *D*-lactate and skeletal muscle strength in middle-aged and older females; Figure S1: Associations among intestinal permeability, GM and HGSW in middle-aged and older males in the decision tree model; Figure S2: Associations among intestinal permeability, GM and HGSB in middle-aged and older males in the decision tree model; Figure S3: Associations among intestinal permeability, GM and HGSW in middle-aged and older females in the decision tree model; Figure S4: Associations among intestinal permeability, GM and HGSB in middle-aged and older females in the decision tree model.
