# Supplementary material for: Intestinal Permeability Associated with the Loss of Skeletal Muscle Strength in Middle-Aged and Older Adults in Rural Area of Beijing, China
Source: Healthcare (Basel). 2022 Jun 13;10(6):1100. doi: 10.3390/healthcare10061100 (PMC9223217; doi:10.3390/healthcare10061100)
Supplement: Supplementary file 1 [file healthcare-10-01100-s001.zip › healthcare-1719883-supplementary.pdf]

Supplementary Table S1. Subgroup analysis of general characteristics of males by age

Characteristic	Middle-aged males N=33	Older males N=14	*P* value
Age (y）	56.5±5.0	68.9±4.1	<0.001
Body mass (kg)	74.1±8.7	64.9±13.1	0.007
BMI (kg/m^2)	25.8±2.6	23.6±3.8	0.032
PBF (%)	26.3±5.5	25.0±6.2	0.495
ASMI (kg/m^2)	7.6±1.2	7.0±0.9	0.023
HGS (kg)	40.8±8.9	32.2±4.4	<0.001
HGSW	0.6±0.1	0.5±0.1	0.185
HGSB	1.6±0.4	1.4±0.2	0.015
Daily exercise level			
Light (n, %)	1 (3.0)	1 (7.1)	0.473
Moderate (n, %)	17 (51.6)	5 (35.8)	
Vigorous (n, %)	14 (42.4)	7 (50.0)	
No response (n, %)	1 (3.0)	1 (7.1)	
Chronic disease			
Hypertension (n, %)	16 (48.5)	9 (64.3)	0.321
T2D (n, %)	5 (15.2)	4 (28.6)	0.419

Quantitative data are shown as mean±SD, categorical data are shown as n (%). Abbreviations: BMI-Body mass index; PBF-Percent of body fat; ASMI-Appendicular skeletal muscle mass index; HGS-Handgrip strength; HGSW-Handgrip strength adjusted by body mass; HGSB-Handgrip strength adjusted by BMI; T2D-Type 2 diabetes.

Supplementary Table S2. Subgroup analysis of general characteristics of females by age

Characteristic	Middle-aged females N=41	Older females N=4	*P* value
Age (y）	55.3±3.8	67.5±2.6	<0.001
Body mass (kg)	65.3±9.1	62.4±10.1	0.554
BMI (kg/m^2)	25.9±3.3	23.6±4.0	0.213
PBF (%)	36.7±8.4	32.6±3.7	0.169
ASMI (kg/m^2)	6.7±0.6	6.3±1.0	0.182
HGS (kg)	27.4±4.4	29.6±5.2	0.366
HGSW	0.4±0.1	0.5±0.1	0.239
HGSB	1.1±0.2	1.3±0.3	0.086
Daily exercise level			
Low (n, %)	0 (0.0)	0 (0.0)	0.569
Middle (n, %)	9 (22.0）	0 (0.0)	
High (n, %)	32 (78.0)	4 (100.0)	
No response (n, %)	0 (0.0)	0 (0.0)	
Chronic disease			
Hypertension (n, %)	16 (39.0)	3 (75.0)	0.295
T2D (n, %)	7 (17.1)	1 (25.0)	0.557

Quantitative data are shown as mean±SD, categorical data are shown as n (%). Abbreviations: BMI-Body mass index; PBF-Percent of body fat; ASMI-Appendicular skeletal muscle mass index; HGS-Handgrip strength; HGSW-Handgrip strength adjusted by body mass; HGSB-Handgrip strength adjusted by BMI; T2D-Type 2 diabetes.

Supplementary Table S3. Correlations among indicators of intestinal permeability and skeletal muscle strength in middle-aged and older adults

Indicator of intestinal permeability	Age	HGS		HGSW		HGSB	
		r	*P*	*r*	*P*	*r*	*P*
DAO	Middle-aged	-0.345	0.003	-0.310	0.007	-0.336	0.003
	Older	-0.313	0.206	-0.198	0.431	-0.228	0.363
LPS	Middle-aged	0.102	0.389	0.092	0.436	0.075	0.526
	Older	-0.303	0.222	-0.028	0.913	-0.256	0.306
D-lactate	Middle-aged	-0.045	0.705	-0.084	0.476	-0.075	0.527
	Older	-0.215	0.391	-0.031	0.904	-0.138	0.586

Abbreviations: DAO-Diamine oxidase; LPS-Lipopolysaccharide; HGS-Handgrip strength; HGSW-Handgrip strength adjusted by body mass; HGSB-Handgrip strength adjusted by BMI.

Supplementary Table S4. Associations among serum DAO and skeletal muscle strength in middle-aged adults

Model	HGS				HGSW				HGSB			
	β value	95% *CI*		*P* value	β value	95% *CI*		*P* value	β value	95% *CI*		*P* value
Model 1	-2.122	-3.480	-0.764	0.003	-0.023	-0.040	-0.006	0.007	-0.086	-0.142	-0.029	0.003
Model 2	-1.665	-2.880	-0.449	0.008	-0.018	-0.032	-0.003	0.020	-0.063	-0.111	-0.015	0.011
Model 3	-0.535	-1.653	0.583	0.342	-0.006	-0.021	0.008	0.405	-0.020	-0.065	0.025	0.373

Model 1: Crude model. Model 2: Adjusted by age, BMI, ASMI, PBF. Model 3: Adjusted by age, BMI, ASMI, PBF, daily exercise level, hypertension, T2Ds, gender. Abbreviations: HGS-Handgrip strength; HGSW-Handgrip strength adjusted by body mass; HGSB-Handgrip strength adjusted by BMI.

Supplementary Table S5. Associations among serum DAO and skeletal muscle strength in older adults

Model	HGS				HGSW				HGSB			
	β value	95% *CI*		*P* value	*β* value	95% *CI*		*P* value	*β* value	95% *CI*		*P* value
Model 1	-0.658	-1.717	0.400	0.206	-0.008	-0.029	0.013	0.431	-0.024	-0.077	0.030	0.363
Model 2	-0.881	-1.929	0.168	0.092	-0.012	-0.029	0.005	0.152	-0.037	-0.079	0.005	0.078
Model 3	-0.941	-2.758	0.875	0.260	-0.006	-0.031	0.019	0.578	-0.039	-0.112	0.033	0.241

Model 1: Crude model. Model 2: Adjusted by age, BMI, ASMI, PBF. Model 3: Adjusted by age, BMI, ASMI, PBF, daily exercise level, hypertension, T2Ds, gender. Abbreviations: HGS-Handgrip strength; HGSW-Handgrip strength adjusted by body mass; HGSB-Handgrip strength adjusted by BMI.

Supplementary Table S6. Associations among serum LPS and skeletal muscle strength in middle-aged and older males

Model	HGS				HGSW				HGSB			
	β value	95% *CI*		*P* value	*β* value	95% *CI*		*P* value	*β* value	95% *CI*		*P* value
Model 1	11.141	-69.855	92.136	0.783	0.278	-0.709	1.265	0.573	0.412	-2.818	3.641	0.799
Model 2	10.167	-52.369	72.704	0.744	0.249	-0.576	1.075	0.545	0.327	-2.197	2.851	0.795
Model 3	7.088	-60.601	74.777	0.833	0.188	-0.701	1.077	0.671	0.188	-2.537	2.913	0.890

Model 1: Crude model. Model 2: Adjusted by age, BMI, ASMI, PBF. Model 3: Adjusted by age, BMI, ASMI, PBF, daily exercise level, hypertension and T2Ds. Abbreviations: HGS-Handgrip strength; HGSW-Handgrip strength adjusted by body mass; HGSB-Handgrip strength adjusted by BMI.

Supplementary Table S7. Associations among serum LPS and skeletal muscle strength in middle-aged and older females

Model	HGS				HGSW				HGSB			
	β value	95% *CI*		*P* value	*β* value	95% *CI*		*P* value	*β* value	95% *CI*		*P* value
Model 1	27.879	-14.257	70.014	0.189	0.300	-0.498	1.098	0.452	0.717	-1.426	2.860	0.504
Model 2	28.447	-15.995	72.889	0.203	0.451	-0.240	1.142	0.195	0.926	-0.859	2.711	0.301
Model 3	38.683	-5.019	82.385	0.081	0.572	-0.120	1.263	0.102	1.292	-0.494	3.078	0.151

Model 1: Crude model. Model 2: Adjusted by age, BMI, ASMI, PBF. Model 3: Adjusted by age, BMI, ASMI, PBF, daily exercise level, hypertension and T2Ds. Abbreviations: HGS-Handgrip strength; HGSW-Handgrip strength adjusted by body mass; HGSB-Handgrip strength adjusted by BMI.

Supplementary Table S8. Associations among serum *D*-lactate and skeletal muscle strength in middle-aged and older males

Model	HGS				HGSW				HGSB			
	β value	95% *CI*		*P* value	β value	95% *CI*		*P* value	β value	95% *CI*		*P* value
Model 1	-2.966	-12.918	6.986	0.551	-0.075	-0.195	0.044	0.212	-0.278	-0.668	0.111	0.157
Model 2	1.788	-6.425	10.002	0.663	0.035	-0.074	0.143	0.523	0.060	-0.272	0.391	0.718
Model 3	3.625	-5.459	12.709	0.424	0.062	-0.057	0.181	0.299	0.137	-0.229	0.503	0.454

Model 1: Crude model. Model 2: Adjusted by age, BMI, ASMI, PBF. Model 3: Adjusted by age, BMI, ASMI, PBF, daily exercise level, hypertension and T2Ds. Abbreviations: HGS-Handgrip strength; HGSW-Handgrip strength adjusted by body mass; HGSB-Handgrip strength adjusted by BMI.

Supplementary Table S9. Associations among serum *D*-lactate and skeletal muscle strength in middle-aged and older females

Model	HGS				HGSW				HGSB			
	β value	95% *CI*		*P* value	β value	95% *CI*		*P* value	β value	95% *CI*		*P* value
Model 1	-1.476	-6.943	3.990	0.589	0.020	-0.082	0.122	0.697	0.078	-0.195	0.352	0.567
Model 2	-0.467	-6.301	5.366	0.872	-0.013	-0.104	0.078	0.773	0.027	-0.206	0.259	0.818
Model 3	0.196	-5.725	6.117	0.947	0.002	-0.091	0.095	0.962	0.056	-0.182	0.294	0.636

Model 1: Crude model. Model 2: Adjusted by age, BMI, ASMI, PBF. Model 3: Adjusted by age, BMI, ASMI, PBF, daily exercise level, hypertension and T2Ds. Abbreviations: HGS-Handgrip strength; HGSW-Handgrip strength adjusted by body mass; HGSB-Handgrip strength adjusted by BMI.

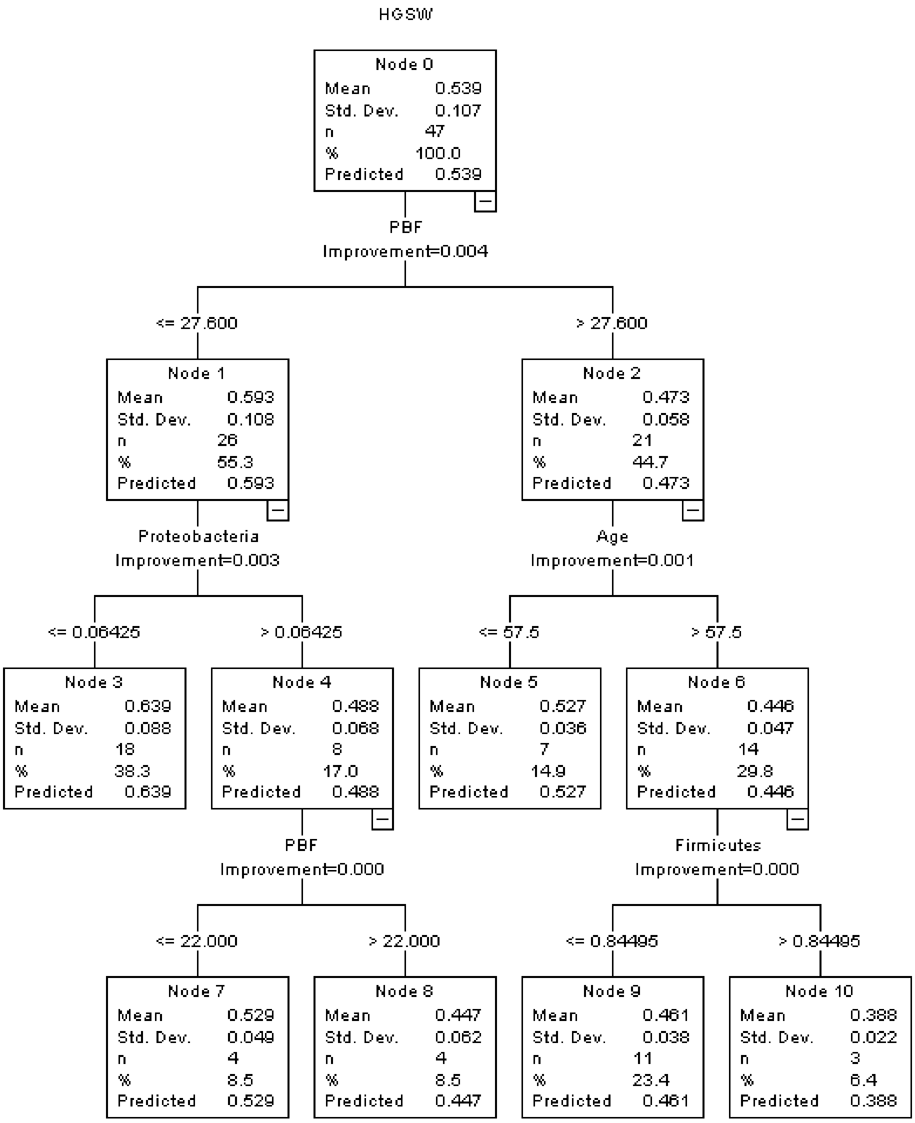

Supplementary Figure S1. Associations among intestinal permeability, GM and HGSW in middle-aged and older males in the decision tree model

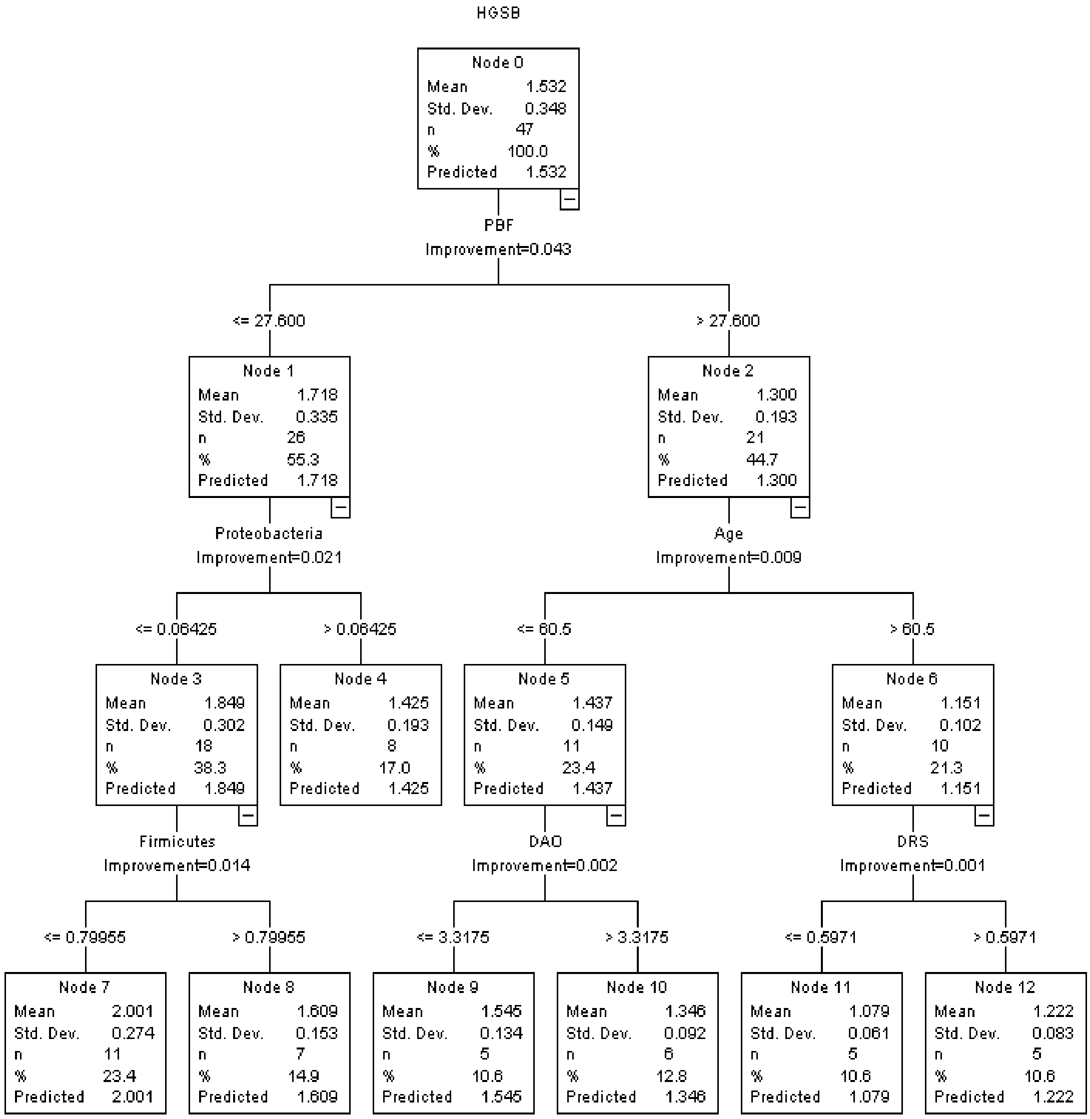

Supplementary Figure S2. Associations among intestinal permeability, GM and HGSB in middle-aged and older males in the decision tree model

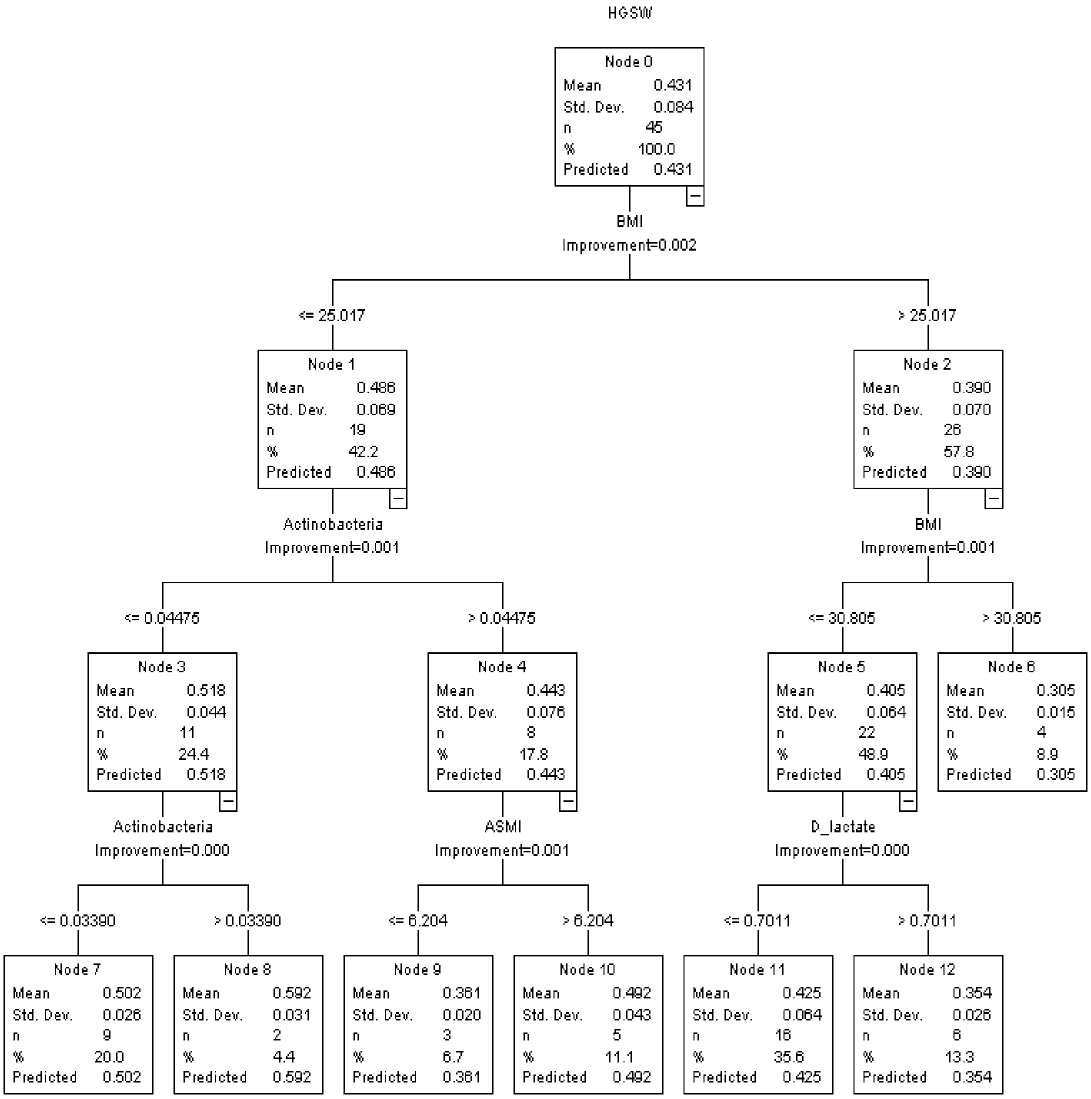

Supplementary Figure S3. Associations among intestinal permeability, GM and HGSW in middle-aged and older females in the decision tree model

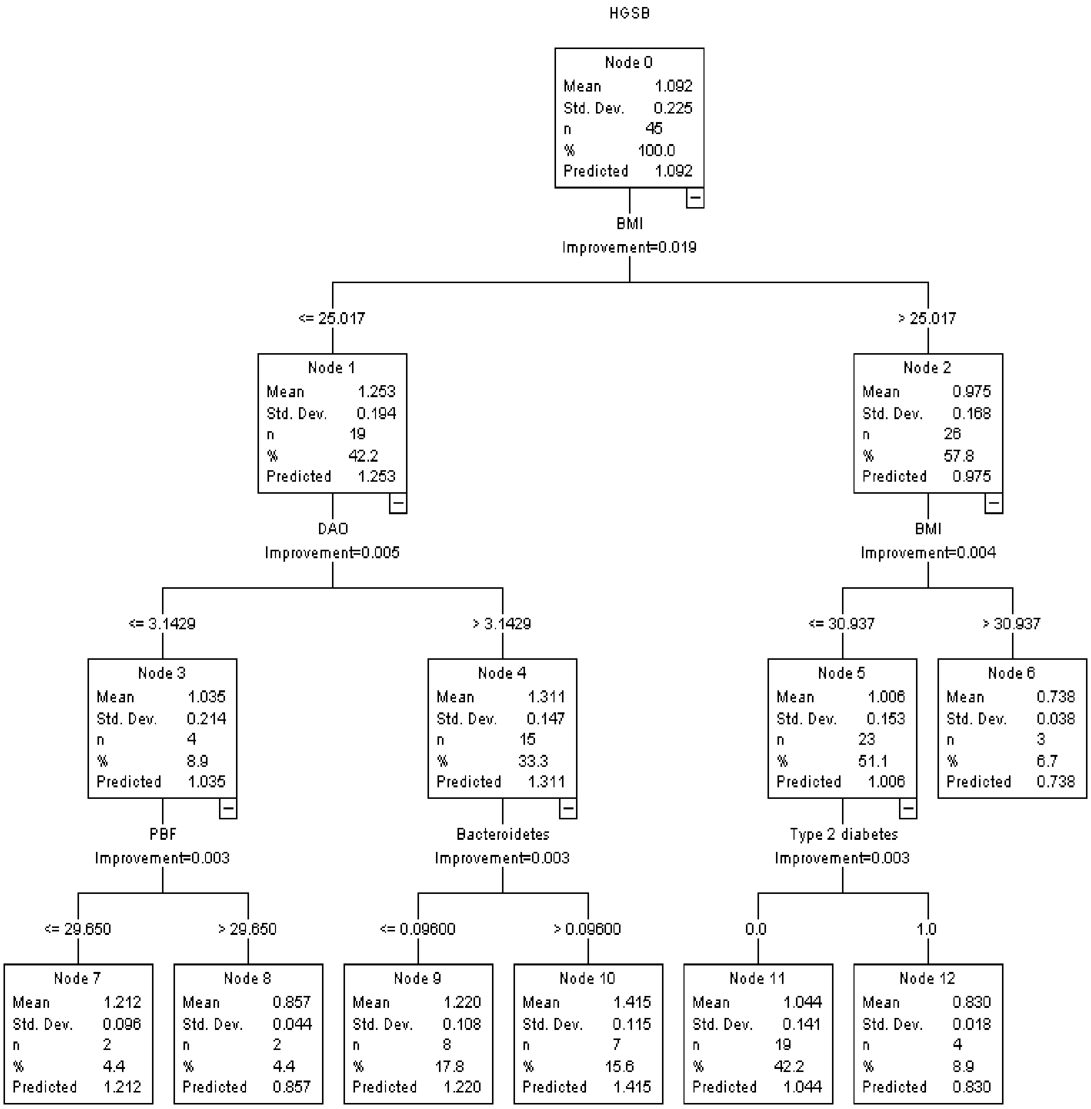

Supplementary Figure S4. Associations among intestinal permeability, GM and HGSB in middle-aged and older females in the decision tree model
